# PSYCHOLOGICAL PROFILE OF PATIENTS ELIGIBLE FOR BARIATRIC SURGERY

**DOI:** 10.1590/0102-6720201600S10008

**Published:** 2016

**Authors:** Graziela Aparecida Nogueira de Almeida RIBEIRO, Helenice Brizolla GIAMPIETRO, Lídia Barbieri BELARMINO, Wilson SALGADO-JÚNIOR

**Affiliations:** Bariatric Surgery Clinic, Clinical Hospital of the Faculty of Medicine of Ribeirão Preto, University of São Paulo, Ribeirão Preto, SP, Brazil

**Keywords:** Psychological evaluation, Bariatric surgery, Depression, Anxiety, Binge eating disorder

## Abstract

**Background:**

The psychologist who works in bariatric surgery has a role to receive, evaluate, prepare and educate the patient who will undergo the surgical procedure. Psychological evaluation becomes important in so far as allows us to obtain data on personal and familiar history and allow tracing of possible psychopathology.

**Aim:**

To collect data on psychological evaluations of patients in a bariatric surgery service of a public hospital in order to describe the psychological profile of patients in this service.

**Method:**

Data were collected from 827 patients between 2001 and 2015, using data from an interview, Beck Depression Inventory (BDI), Beck Anxiety Inventory (BAI) and Binge Eating Scale (BES).

**Results:**

The mean age of patients before surgery was 39 years+/- 10, the mean BMI was 51 kg/m²+7, and most patients (81%) were female. The average score on the BDI was 14.8+8 and women had significantly higher scores than men. On the BAI the average score was 11+8 and on the ECAP was 14+8, both with no difference between groups.

**Conclusions:**

Psychosocial characteristics of the patients points to the significant presence of indicators of depression, with low levels of anxiety and binge eating.

## RESUMO

**Racional:**

O psicólogo que atua na área da cirurgia bariátrica tem papel de acolher, avaliar, preparar e conscientizar o paciente que vai ser submetido ao procedimento cirúrgico. A avaliação psicológica reveste-se de importância na medida em que obtém dados sobre história pessoal e familiar, além do rastreio de possíveis alterações psicopatológicas.

**Objetivo:**

Analisar as avaliações psicológicas dos pacientes de um serviço de cirurgia bariátrica de um hospital público, buscando traçar o perfil psicológico dos pacientes deste serviço.

**Métodos:**

Foram coletados dados de 827 pacientes entre 2001 e 2015, utilizando-se dados de uma entrevista, Inventário Beck de Depressão (BDI), Inventário Beck de Ansiedade (BAI) e Escala de Compulsão Alimentar Periódica (ECAP).

**Resultados:**

A idade média dos pacientes antes da operação foi 39 anos+10 e o IMC médio foi 51 kg/m²+7, sendo a maioria (81%) do gênero feminino. A pontuação média no BDI foi 14,8+8 e as mulheres tiveram pontuação significativamente maior que os homens. No BAI a pontuação média foi 11+ 8 e no ECAP 14+ 8, ambos sem diferença entre os grupos.

**Conclusão:**

A caracterização psicossocial dos pacientes avaliados aponta para a presença significativa de indicadores de depressão, com baixos níveis de ansiedade e de compulsão alimentar.

## INTRODUCTION

Obesity prevalence, especially obesity grade 2 and grade 3, has been increasing dramatically in the last three decades all over the world^13^ ^,^ ^18^. Despite the encouragement changes concerning lifestyle and diet for obesity grade 3, weight loss in general is not enough to promote a better physical health. In this cases bariatric surgeries poses as the best choice for treatment as long as it favors significantly and sustainably weight loss associated to the improvement of the comorbidities^5^ ^,^ ^6^.

To be eligible for the surgery it is mandatory that the patient is evaluated by a multidisciplinary team, including physicians from different specialties, psychologist, nutritionist, and others^6^ ^,^ ^4^.

The psychologist in the area of bariatric surgery has the role to receive, evaluate, prepare and bring the patient awareness to the fact of undergoing a surgical procedure. The psychological evaluation is very important in the sense of gathering data on family and personal history as well as the patient's weight, besides screening possible psychological disorders. The more is known about the patients more possibility of offering a better and suitable support to the patients demands both in pre- and postoperative stage^6^.

The investigation of possible associations between psychological disorders and obesity grade 3 has been very frequent in the last few years, among them we can mention: depression, anxiety and binge eating disorders, particularly in the patients eligible for bariatric surgery^8^ ^,^ ^14^ ^,^ ^15^ ^,^ ^21^ ^,^ ^25^.

However, in studies conducted in Brazil there are divergent approaches concerning the presence of psychological disorders more specifically anxiety and depression. Most of the studies in this area are very restricted in relation to the number of participants in the research, what makes it difficult to the generalize^17^ ^,^ ^20^ ^,^ ^24^.

Considering this gap in research on psychological aspects of bariatric surgery services in Brazil, this study aimed to gather data on psychological assessments of patients of bariatric surgery service of a public hospital. 

## METHOD

This research was approved by Ethics Committee from Clinical Hospital of the Faculty of Medicine of Ribeirão Preto of the University of São Paulo in accordance to the process 8763/2009.

It is a retrospective study with a quantitative approach conducted by psychologists from the bariatric surgery team of Clinical Hospital of the Faculty of Medicine of Ribeirão Preto of the University of Sao Paulo. All the completed psychological evaluation was included in this study, totaling 827 preoperative evaluations.

### Participants

Data of 827 patients were consecutively evaluated between 2001 and 2015 in the bariatric surgery service of a public hospital. All patients were eligible to bariatric surgery but more specifically Roux-en-Y gastric bypass. All the evaluation was part of the presurgery preparation routine.

### Instruments

The following instruments were applied: a semi-structured interview aimed to collect socio-demographic data; Beck Depression Inventory (BDI)^7^ and Beck Anxiety Inventory (BAI)^7^ that aimed to investigation symptoms of depression and anxiety respectively; and the Binge Eating Scale (BES)^11^ was applied to evaluate the symptoms of binge eating disorder.

The score for the three instruments (BDI, BAI and BES) allowed the classification the symptoms in different levels of intensity, through the total score.

The BDI consists in 21 items including symptoms and behavior which intensity varies from 0 to 3. The level of depression is classified according to the total score obtained: from 0 to 11=minimum or no depression, from 12 to 19=mild, from 20 to 35=moderate and from 36 to 63=severe. The cut-off point considered is 12.

The BAI consists in 21 items each of them contains four possible alternatives in different crescent grade in each symptom. The level of anxiety is classified as: from 0 to 10=minimum or no anxiety, from 11 to 19=mild, from 20 to 30=moderate, from 31 to 63=severe. The cut-off considered is 11.

The BES consists in 16 items. The level of binge eating is classified according to the score obtained. Patients who score <17 are considered as no binge eating; the score between 18 and 26 as moderated binge eating, and the ones with scores >27 are considered as severe binge eating. The cut-off point considered is 17. 

### Statistical analysis

The statistical analysis of sociodemographic variables related to age and BMI were performed using median and standard deviation. Statistical treatment was proceeded applying the t-test for independent sample aimed to verify the difference between the genders. The categorical data was presented by the frequency and percentage. The data related to the BDI, BAI and BES instruments were codified in accordance to the specific recommendation of each one of them, continuing the statistical treatment applying the Chi-square test. In order to compare the results in BDI, BAI and BES with the normative sample t-test was done. All of the statistical analyses were performed using the SPSS 17.0 package. p-values <0.05 were considered statistically significant.

## RESULTS

Of the 827 participants, 669 (81%) were female and 158 (19%) male. The average age of participants was 39.5+10 years and the mean BMI was 51.1+7 kg/m², without significant differences between genders (Table 1).

**TABLE 1:** Characterization of the sample according to age and BMI (n=827) - mean values and standard deviation

	Total	Female	Male	p (t-test for independent sample
	M (SD)	M (SD)	M (SD)	
Age (years)	39.5 (+10)	39.7 (+10)	38.5 (+10)	0.20
BMI (kg/m²)	51.1 (+7)	50.9 (+7)	51.8 (+7)	0.25

Considering education, it was observed a higher percentage of participants that were between 10 and 12 years of schooling, with a statistically significant difference between genders, showing that the number of years of schooling among men was higher compared to women. Most participants (60.7%) had a partner at the time of evaluation, with no difference between genders. Most of them had paid job. Among men the percentage of those who worked was significantly higher (69%) compared to women (Table 2).

**TABLE 2:** Characterization of the sample according to educational level, marital status and paid job (n=827) - Frequency and percentage values

	Total	Female	Male	p (Chi-square test)
	F (%)	F (%)	F (%)	
Education (years)				
< 9	329 (39.8)	282 (42.2)	47 (30.0)	0.002*
10-12	369 (44.6)	290 (43.3)	79 (50.0)	0.002*
>13	129 (15.6)	97 (14.4)	32 (20.0)	0.002*
Marital status				
No partner	325 (39.3)	253 (37.8)	72 (45.4)	0.09
With partner	502 (60.7)	416 (53.1)	86 (54.6)	0.09
Paid job				
No job	363 (43.9)	314 (46.9)	49 (31.0)	0.001*
With job	464 (56.1)	355 (53.1)	109 (69.0)	0.001*

* Statistically significant difference

According to the evaluation by the instruments, on the BDI the average total score was 14.8+8 points, showing a statistically significant difference between groups. In this case, the score on the BDI was statistically higher among women compared to men. Regarding the BAI, the average score was 11+8, with no statistically significant difference between groups. As for the BES, the average score was 14+8, also with no statistically significant difference between groups (Table 3).

**TABLE 3:** Score in BDI, BAI and BES - mean values and standard deviation

	Total	Female	Male	p (t-test for independent sample
Instruments	M (SD)	M (SD)	M (SD)	
BDI	14.8 (+ 8)	15.1 (+ 8)	13.3 (+ 6)	< 0.005*
BAI	11.0 (+8)	11.1 (+ 8)	10.5 (+ 9)	0.45
BES	14.4 (+8)	14.6 (+8)	13.5 (+7)	0.15

* Statistically significant difference

Considering the rankings in BDI, BAI and BES and according to the specific technical recommendations for each one, it was observed the percentage of participants who had or not the presence of disorders (Table 4).

**TABLE 4:** Frequency and percentage of participants relating to different classification in the instruments BDI, BAI and BES (n=827)

	Total	Female	Male	p (Chi-square test)
Instruments	F (%)	F (%)	F (%)	
BDI (classification)				
Minimum	322 (38.9)	251 (37.5)	71 (45.0)	0.03*
Mild	300 (36.3)	239 (35.7)	61 (38.6)	0.03*
Moderate	183 (22.2)	157 (234)	26 (16.4)	0.03*
Severe	22 (2.7)	22 (3.3)	--	0.03*
BAI (classification)				
Minimum	474 (57.3)	375 (56.0)	99 (62.9)	0.04*
Mild	229 (27.7)	186 (27.8)	43 (27.1)	0.04*
Moderate	100 (12.1)	91 (13.6)	9 (5.7)	0.04*
Severe	24 (2.8)	17 (2.5)	7 (4.3)	0.04*
BES (classification)				
No binge eating	566 (68.4)	449 (67.1)	117 (74.1)	0.14
Moderate binge eating	190 (22.9)	157 (23.5)	33 (20.9)	0.14
Severe binge eating	71 (8.6)	63 (9.4)	8 (5.0)	0.14

* Statistically significant difference between groups

Thus it was found that the BDI, when considering the scores at all levels men achieved a statistically higher score compared to women in minimal and mild depression ratings. On the other hand, women had statistically higher scores in moderate and severe depression ratings. In other words, men showed fewer symptoms of depression than women.

Regarding BAI, significant differences between the groups were observed, pointing to higher scores on ratings of minimum anxiety and severe anxiety among men. On the other hand, women obtained higher scores in the moderate anxiety rating.

In BES significant differences between groups were observed. However, men showed less indicators of the presence of binge eating compared to women who obtained scores higher in mild and severe binge eating ratings.

Comparing the rate obtained in the three instruments BDI, BAI and BES with the average used by the cut-off rate is observed that in the comparison with BDI, the average obtained by the participants was significantly higher showing higher rate of depression. In relation to the BES the average rate obtained by the participants was significantly lower when compared to the normative sample showing lower rates of binge eating disorders (Table 5).

**TABLE 5:** Comparison of the average score in the instruments BDI, BAI and BES reached by the participants in comparison to the cut-off points of the respective instruments

	Total	Average normative sample (obesity)	p (t-test for independent sample)
Instruments	M (SD)	M	
BDI	14.8 (+ 8)	12	< 0.001*
BAI	11.0 (+8)	11	< 0.92
BES	14.4 (+8)	17	< 0.001*

* Statistically significant difference

## DISCUSSION

The social-demographic profile of the participants showed the prevalence of obesity grade 3 in women that were looking for bariatric surgery. This data is in accordance to other studies which also observed such prevalence^3^ ^,^ ^10^ ^,^ ^23^. Some studies also try to explain this fact as a result of female predisposition of accumulating fat, as well as for their economic and cultural limitation, that impair their access to health services and the experience of a more favorable lifestyle through the practice of physical activities and oriented diets^9^. It's also necessary to consider the psychosocial aspects. Women have been suffering a great deal with the obesity stigma of thinness in the sense that it falls on them the beauty stereotype socially constructed and perpetuated in society, demanding an idealistic pattern of beauty^22^.

The average age of the participants was 39.5 years old, showing a young population in reproductive age and also economically active. In this matter it's necessary to make some considerations. The fact of having more women looking for the surgery added up to the fact that the majority of them being in reproductive age show the necessity of general health services make all efforts to give orientation concerning the possibility of pregnancies after the surgery, aiming to minimize the negative impacts of that an unplanned pregnancy can cause to the woman as well as to the baby.

The population studied was characterized by the prevalence of economic active people and the ones that had a partner, what can indicate the psychosocial protection in the sense that the patients seem to be making an adequate use of their affective and cognitive resources favoring lower levels of anxiety indicators^1^. In other words considering the fact that these people are coming to a time that precedes the long-awaited and important surgery, the level of anxiety evaluated by BAI was similar to the normal population in contrast to other studies that also evaluated the presence of anxiety before surgery and found very high levels of anxiety^8^ ^,^ ^25^.

In the evaluation referring to BDI a higher score was observed related to some degree of depression (mild, moderate and severe) both in men and women. The higher scores found were different from other studies that also used BDI as a screening instrument of preoperative depression showing the needed of adequate psychological support to the emotional demands that had emerged in this population, favoring an emotional strengthening. Studies about depression show its prevalence in women what is confirmed in this study. However, patients that are employed would have the tendency to show lower scores of depression. In this study, the analysis of the incidence of depression in those that had an occupation or not weren't performed. Nevertheless, higher scores of depression are observed showing significant damage in this population. This study makes clear the importance and necessity to have follow- up studies in order also to understand in the postoperative the emotional dynamic of these patients that have already significant emotional suffering in the preoperative^2^ ^,^ ^3^. Besides that, there is a clear need to investigate which psychosocial aspects of these patients that contributed to the emotional damage that has taken or favored the development the significant depression symptoms.

A finding in this study refers to lower scores verified in BES. Studies that relate obesity and binge eating show the high prevalence of the binge eating, especially among eligible patients to bariatric surgery^12^ ^,^ ^19^. Besides that, studies show the association between binge eating and high levels of depression symptoms, what was not observed in this study^16^. One of the reasons for lower scores in the BES refers to the fact that these patients that were waiting for the surgery were already receiving nutritional support, once they started the treatment in bariatric surgery service. All of them had to go through dietary re-education aiming an adequate diet already considering the bariatric surgery. It was verified the benefit that these patients obtained by having such care and orientation favoring the experience of healthier eating habits. However, besides that, it's necessary to consider the hypothesis of these patients that received support might have answered the BES questionnaire trying to give "the better answer" or "the most adequate answer" but not really experiencing an internal change. The answer for this question can only be given through the follow-up study investigating how these patients are in the postoperative stage in the emotional point of view, as well as the eating habits behavior. 

It is expected that a broad study like this can be a reference to other bariatric surgeries services that aim to understand the psychological aspects associated with bariatric surgery patients. 

It is important to consider that the data presented in this study refers to a part of the psychological evaluation performed on the service in question since it is a very comprehensive assessment in which the information gathered would go beyond the objectives of this study.

The psychological evaluation before bariatric surgery has its importance in the sense that it allows the discrimination of the psychosocial aspects of the eligible patients of bariatric surgery. It also allows the team to work in the prevention of future problems and to work in the attention to the neuralgic aspects that need more attention and care and can also contribute to improving the chances of success in the surgery.

## CONCLUSIONS

Psychosocial characterization of patient evaluation shows a significant indicator of depression with lower levels of anxiety and binge eating.
